# Patent Acquisitions in the Healthcare Industry: An Analysis of Learning Mechanisms

**DOI:** 10.3390/ijerph20054100

**Published:** 2023-02-24

**Authors:** Carlo Giglio, Gianluca Salvatore Vocaturo, Roberto Palmieri

**Affiliations:** 1 Department of Civil, Energy, Environmental and Material Engineering, Mediterranean University of Reggio Calabria, Via Zehender, Località Feo di Vito, 89122 Reggio Calabria, Italy; 2 Department of Mechanical, Energy and Management Engineering, University of Calabria, Via Pietro Bucci, Cubo 46/C, Ponte Carrabile, 87036 Rende, Italy

**Keywords:** patents, patent acquisition, knowledge acquisition, learning from patents, healthcare industry

## Abstract

The healthcare industry at large is used as a case study to suggest a methodological technique for evaluating patent citation networks to analyze cross-country creativity/knowledge flows. It intends to provide insight on the following research issues: (a) how to examine cross-national creative/learning flows; and (b) have nations with present patent owners profited from patent acquisitions? The research field at hand is currently under-explored, justifying the motivation for conducting this investigation, even though it has economic relevance in innovation patterns worldwide. The analysis of over 14,023 firms has shown that: (a) owners have acquired patents across borders, and (b) acquired patents (granted between 2013 and 2017) are cited by later patents (2018–2022). The methodology and findings are transferable to other industries. They can be used by managers and policymakers to (a) assist businesses in predicting innovation trajectories and (b) assist governments in designing and putting into action more effective policies that foster patented innovations in sectors that are deemed to be relevant to the national interest, thanks to the adoption of a new, complementary theoretical viewpoint that merges the micro- and macro-economic perspectives of citation flows.

## 1. Introduction

In order to determine if and how information flows were helpful with respect to the scope and intensity of knowledge dissemination, analysis of patent citations and the associated networks has been widely utilized (and continues to be used) [1,2,3,4,5,6,7], while in the existing literature on patent citations, creativity flows are not at all taken into account. An (often implicit) requirement mentioned in the majority of this research is that applicants for citing and referenced patents are different. In other words, measuring knowledge dissemination has been the primary goal of tracking patent citation flows. However, a very low number of specialized research efforts have concentrated on the knowledge diffusion characteristics that distinguish particular technological sectors, i.e., knowledge pathways and decay, and procedural quirks of patent offices around the world, i.e., patent office bias. Following earlier reference research, [8] demonstrated that both elements are relevant when considered independently [9,10]. Our study, which is consistent with accepted methods in the literature, focuses on technological areas that are specifically relevant to the healthcare industry, based on both the International Patent Classification (I.P.C.) and Cooperative Patent Classification (C.P.C.) [11,12].

A ground-breaking study conducted in the last 20 years addressed the question of the geographic and technological reach of copyrighted ideas, which is a different area of investigation in the literature (e.g., [13,14,15,16,17,18,19]. Inventors frequently cite same-country patent applicants, ignoring potentially more useful cross-national citations. Self-citations are also often used, which raises the number of citations from the same government. Because self-citations and duplicate country citations are linked to a country’s technological scenario, inter-nation citations between citing and cited inventors are further diminished. As a result, this may impact at the national level both the level of openness and growth of the relevant technological sector, and significantly influence corporate competitiveness. This statement highlights the importance of investigating the effect of the national source because of the dual purpose of (a) giving managers a competitive forecasting tool to determine which environments are favourable for creativity- and knowledge-based innovations and (b) assisting policymakers in developing and implementing the right policies to foster technological improvements and the industry’s competitiveness for the country. Such a dual purpose is pursued by means of a focus on patent acquisitions data, based on a fourfold literature-grounded choice explained in Section 2.

Following the introduction, Section 2 reviews the relevant references on cross-national patent citation flows and discusses the reasons behind the paper. Section 3 then describes the data collection and methodological aspects. Section 4 then presents the results and discussions, and Section 5 concludes.

## 2. Literature Review and Motivation

The current literature demonstrates a dual focus on macroeconomics and microeconomics [1,8]. The former highlights variables impacting on economic growth and industry competitiveness by analyzing knowledge flows between applicants from various nations [15,20,21,22,23,24,25,26,27,28]. The term “knowledge interactions” refers to various additional activities that affect a nation’s macroeconomic outlook, such as social interactions, public–private partnerships, and joint industry collaborations [20]. Finally, by combining and taking advantage of the synergistic resources of national innovation systems, they all assist in obtaining more significant economies of scale and direct national innovation and technological advancement in the direction of global technological frontiers [20,25,27,29,30,31,32,33]. Therefore, macroeconomic agglomerates, such as countries and regions, may be rated, and their technological potential (and associated advancements and trajectories) could be improved by the implementation of suitable policies [27,34,35,36,37,38,39]. Moreover, firm-level factors affecting competitiveness of patent values also apply [1,28,37,40,41,42,43,44].

These findings demonstrate the necessity of combining macroeconomic and microeconomic perspectives to ensure a thorough understanding of the effects of citation flows on companies and public policies.

The topic of patent acquisition has been covered in earlier research [45,46,47,48,49], while other studies cover licensing [50], pricing [51], auctions [50,51,52,53,54,55,56] and reassignments [57,58,59,60,61]. However, another quota of studies in this area focused specifically on patent citations and patent citations flows and their relationships with knowledge and innovation mechanisms: a set of examples, showing how patent citation flows are linked to higher knowledge diffusion, knowledge absorption (e.g., learning mechanisms), innovation/patenting activities, (inter-)countries’ or (inter-)industries’ innovation performances, is provided by [47,48,49,62,63,64,65,66,67,68,69,70,71,72,73,74,75,76]. It is worth noticing that “*an illustrative analysis examining the magnitude and direction of measurement error bias suggests that measuring knowledge flows with patent citations can lead to substantial underestimation of the effect of public research on firms’ innovative performance*” ([63], page 1), thus, showing the conservative nature of findings on countries’ innovation performances derived from patent citations.

In detail, the existing literature on country-level analyses of knowledge flows investigates the relationship between the intensification and diversification of knowledge flows, on the one side, and the identification of attractive opportunities for innovation, development and catching-up, on the other side, by considering a broad dataset from the USPTO including patents from 1982 to 2006 [62]. Other contributions focus more specifically on knowledge flows measured as backward citations from public research only, identifying some sources of systematic errors in the measurement linked to the utilization of backward citations for both patents and non-patent references [63], as their combined use has increased significantly in the last two decades [64]. In fact, 73% of papers cited by USA industry patents are generated by “*public science, authored at academic, governmental, and other public institutions*”, while less than one third are authored by industrial scientists ([76], page 1). Moreover, citations provide an indication of S&T precedents and, then, they represent knowledge that can be tracked [64]. Moreover, the topic of the value of patents is linked to the number of citations as well, since the higher the number of references and citations to prior art, the higher the value of such S&T precedents [65]. Past literature links patent citation studies as well as patents themselves to an organization’s life chances, whilst other studies on knowledge flows across countries are linked to differences among examiners’ and applicants’ citations or to the impact of crises on the national (e.g., Japanese) innovation capacity, also proposing patent citations as an overall approach in order to build technology indicators at large and capture knowledge and trends on technology development and performance [65,66,67,68,69,70,71]. Cross-country cross-sector analyses have evaluated knowledge spillovers “*within and across sectors and national boundaries using European patents and citations for a large group of OECD countries occupying different positions with respect to the world technological frontier*”, whereas “*the magnitude and relative importance of national and international spillovers is estimated accounting for the role of prior research experience in providing the necessary skills for technology adoption and further development, controlling for potential endogeneity and feedback effects*” ([71], page 1). The findings in [71] suggest that “*international spillovers are effective in increasing innovative productivity in laggard countries, while technological leaders are a source, rather than a destination, of knowledge flows. The paper then presents quantitative estimates of the effect of absorptive capacity on innovative performance, through knowledge spillovers, and shows that absorptive capacity increases the elasticity of a laggard country’s innovation to international spillovers, while its marginal effect is negligible for countries at the technological frontier*” ([71], page 1). Moreover, the combination of patent citation studies with the topics related to absorptive capacity and knowledge spillovers is key in order to assess the role of prior R&D experience in enhancing a country’s ability to understand and improve upon external knowledge [72]. In particular, a cross-country cross-sector analysis based on patents and citations is important in order to prove that “*absorptive capacity increases the elasticity of a laggard country’s innovation to international spillovers, while its marginal effect is negligible for countries at the technological frontier*” ([72], page 1). Other studies have developed an innovation patent index in order to measure the innovation performance of firms based on secondary, patent-related data [73]. The five dimensions of this analysis are: efficiency, time, diversification, quality, and internationalization; such dimensions were developed through three machine learning algorithms and a literature review, whereas patent forward citations are the proxy of innovation performance in firms [73]. Such a performance indicator appears as a very usable and fast tool for both mangers and policy-makers aiming at planning future activities and supporting innovation capabilities in firms and organizations at large [73]. In [74], patent citation studies are part of a more comprehensive corporate technological performance assessment, that is defined as “*the use of patent counting, clustering, and citation analysis in the evaluation of corporate, industry-wide, and national technological activity*” ([74], page 1). As [75] states, “*patents citation is a developing concept and has gained momentum in recent past. Patents citation contains valuable data and if analyzed well, may sometimes reveal concealed mysteries of the information flow between countries, laboratories, companies, and universities. [...] Patents citation reveals the diffusion of information and its applicability into many other technical fields which give birth to a new technology*” ([75], page 1).

Four factors led to the decision to limit this investigation to acquired patents. First, businesses operating in very competitive environments, such as the one faced by healthcare, tend to obtain primarily strategic patents that they intend to capitalize on. Second, healthcare businesses integrate talent acquisition with patent acquisition because, even though the information is innate in people, the healthcare environment allows patent purchases to be essentially viewed as a stand-in for knowledge acquisitions. Therefore, by owning and controlling patent portfolios and their associated technical trajectories, healthcare enterprises might further develop innate knowledge and competitive advantages. The level of a company’s commitment to influencing the healthcare industry and assuming its technological leadership is also demonstrated by patent acquisitions. Thirdly, the only information our data sources provided are related to patent purchases. Fourth, mixing different data would be methodologically inappropriate and lead to inaccurate conclusions. Data on patent acquisitions should not be mixed with non-acquisition data. Additionally, no healthcare-specific studies have been undertaken to date, which encourages us to concentrate on this area and determine whether or not earlier findings from the literature also apply to this field [8,9,10,11,12,13,14,15,16,17,18,19]. Finally, even though several researches already listed in this section addressed national-level analysis, only a few embraced the international perspective [26]. This serves as more evidence that this work explores a topic that is both important and under-studied in the field of patent citation research.

## 3. Methodological Approach and Data Collection

### 3.1. Sample and Methodology

**Figure 1:**
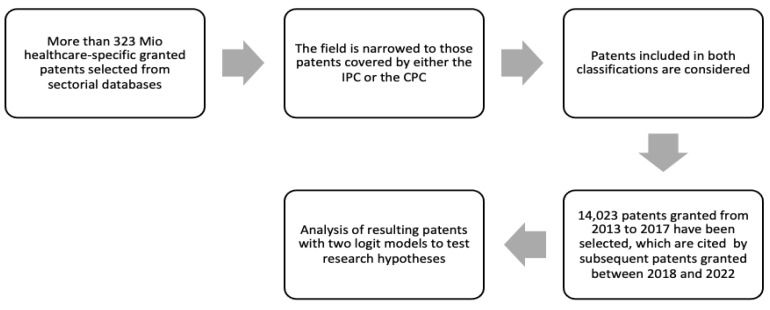
Methodological approach and stages.

The overall approach and the associated processes are outlined in the table below (Figure 1) and are covered in more detail in the following paragraphs. As a presumption, the data processed were extracted from the Bureau Van Dijk (BVD), which combines data from several databases worldwide. BVD’s database generates datasets that are ready to be processed and less exposed to statistical biases, based on extant literature [47,48,49], thus, making Kim and Lee’s [46] data pre-processing unneeded [47,48,49]. In this analysis, 14,023 firms’ healthcare-related patent data are processed according to the steps in Figure 1. From a methodological standpoint, we adopted the consolidated concept of the elasticity of R&D patenting on a 5-year timeline to investigate learning effects, thus, adopting a conservative and more robust approach towards the patent office bias that potentially affects statistical analyses on patents, as described in Section 1 [9,10,11,12,45,77,78,79,80]. The statistical analysis was implemented by using I.B.M.^®^ SPSS^®^ Statistics 27 (IBM, Armonk, NY, USA).

### 3.2. Variables and Measures

We primarily base our study on [37,47,48,81,82] publications in keeping with the body of existing literature. Patents that specifically reference prior art in their applications demonstrate that the latter was in some way helpful in the development of the referencing patent. This dynamic affecting the generation of new patents is represented by the learning flow, as the most recent patent is thought to be developed based on prior (cited) art [47,48]. Instead, those newly filed patents from the acquiring owners that do not refer to the foreign patents they previously purchased demonstrate that these owners still exhibit relevant creativity concerning the subject matter of their acquired patents. Refs. [47,48] assert that earlier innovations consistently offer a certain amount of pertinent, but general knowledge that supports patent development activities. This dynamic is defined as creativity flow. In more detail, creativity takes its place as the primary innovation trigger when previously acquired knowledge is not cited in new patents [37,47,48,82]. A cross-country learning effect occurs when a new patent is developed based on previous patents already acquired from another country [81]. Creativity effects occur when new patents are developed, but do not refer to previous acquired patents [47,48].

However, in this paper we refer to the more comprehensive concept of knowledge flow to track the existence of either a creativity flow or a learning flow, depending on whether the acquired knowledge is directly cited (i.e., explicitly utilized) or just useful (i.e., not cited) in the newly granted patents.

The dependent variable is a binary one measuring the cross-country learning effect, “DepVar” (0 = no cross-country learning, 1 = cross-country learning).

The independent variable measures the number of cross-country patent acquisitions, “IndVar”. Additionally, following [82], we define some control variables for the investigation of knowledge and creativity patterns following [47,48], to look for the firm, technology, and environmental variations. Control variables and descriptive statistics are shown in Table 1a–c.

Binary logistics models are the correct choice for our study, due to the binary dependent variable. Thus, we use two logit models following [82,83].

## 4. Results and Discussion

Multicollinearity was tested through variance inflation factors (VIFs): as they are below 3.5, VIFs prove that this study is not affected by relevant multicollinearity issues [49]. Endogeneity has been treated through the avoidance of conceptual mistakes, the adoption of control variables, and literature-supported hypotheses [49]. Autocorrelation is never an issue in cross-sectional datasets [49]. Heteroskedasticity has been proven as not relevant through White tests I and II, the Breusch–Pagan test, and Newey–West HC3 estimation [49].

In Table 2a,b, we report correlations of the independent variables. Overall, we find a strong association between technology variables and independent variables assessing the frequency of cross-country acquisitions. Table 2b reveals that the analysis at the country level is not significant. This may imply that in the healthcare sector, the length of time required to produce new patents, the number of citations they receive and their inventiveness tend to influence how desirable it is to acquire “foreign patents” from other nations. Likewise, the firm-level control variables are highly associated with one another in both models. This may indicate that large companies in the healthcare sector frequently file numerous applications, even before they purchase any patent from other nations, necessitating a propensity for knowledge absorption. Another argument is how many people in large companies typically offer highly specialized and/or differentiated skills and expertise.

Furthermore, they might have access to more significant resources that support technical innovation processes, and this guarantees such companies an extraordinary ability to update existing information and create new patents. A significant correlation among firm and environment control variables is also discovered. Findings demonstrate how businesses are typically impacted by their surroundings: businesses with high potential for innovation and knowledge-generating solid capabilities are related to national innovation systems that provide a supportive context - especially when dramatic transformations and crises are ongoing - that may help firms turning difficult and uncertain scenarios into opportunities [82,84]. The relatively greater correlation among firm and environment variables in Table 2b compared to Table 2a is explained in this way. This is especially true when creativity is not only deemed as crucial to the national sector at hand, but it is also facilitated.

In conclusion, linkages among firm and environment variables demonstrate that the macroeconomic and microeconomic perspectives of investigation are not separated. As stated in Section 1 and Section 2, this underutilized combined method is one of the driving forces behind our study. Finally, concerning the correlations between levels, our study demonstrates that none of the previously mentioned linkages between the firm and environmental levels have changed. However, there is no collinearity between technology and firm ranks.

Table 3a–d presents the findings from the two logit models. The knowledge and creativity flow studies in Table 3a,c demonstrate a high degree of reliability due to their high percentages of predictability.

The results show that the quota of inter-nation acquisitions positively and significantly affects the cross-country learning variable and also the creative cross-country effect. In contrast, firm and environmental variables do not have any impact. Results indicate that in the healthcare business, the likelihood that present owners file patents mentioning the acquired patents increases as the quota of inter-nations acquisitions increases.

Additionally, the more cross-country patent acquisitions there are, the more likely it is that new patents will be developed creatively (and related products). We thus validate the “learning-by-acquisition” effect, according to which companies who acquire foreign patents relating to healthcare are able to learn and, in turn, to file new patents. We also confirm the impact on creativity, which states that businesses that acquire foreign patents relating to healthcare can continue to exercise relevant creativity and produce innovations unrelated to those patents. We don’t discover any nations with significantly greater creative ability than their rivals. The corresponding environment-level conditions do not favour any location-related effect for firms aiming at developing more creative, radical technologies.

Also, policymakers should consider that new conditions should be implemented in order to foster innovations in healthcare on both the microeconomic and the macroeconomic levels. First, patents developed by firms through cross-country acquisitions foster their own innovation capabilities and competitiveness, but also generate positive effects for the national economy, as highlighted by the extant literature in Section 2 [15,20,21,22,23,24,25,26,28]. Hence, policymakers may evaluate the strategic relevance of a country with reference to the healthcare industry and its global competitiveness and also foresee patterns of technological development, as mentioned in Section 2 [27,34,35,36,37,38,39].

The control variables linked to the technological layer of our model are significant and have a positive effect on cross-country learning mechanisms. By contrast, the remaining control variables are not relevant, except for % of R&D expenditure/turnover and NOPAT, which are clearly linked to the number of strategic efforts devoted towards and of resources available for patented innovations. The importance of technology age and the value of patent citations may indicate that both the amount of time required to produce patents and the number of citations contribute to determining the desirability of patents from abroad. In a nutshell, three factors promote the learning effect: (a) the short time required from the simple acquisition of patents developed abroad until obtaining new patents; (b) the high number of mentions attained by the desirable foreign patent; and (c) the efforts put in innovation activities measured as % of R&D expenditure/turnover and NOPAT.

## 5. Conclusions

The originality of this study can be identified from three perspectives. As for the first one, we develop an in-depth analysis on the learning-by-acquisition effect through patents related to the healthcare sector, whereas we found a less explored research stream, as demonstrated in Section 1 and Section 2, related to technology diffusion dynamics and industry-related spillovers. Thus, this work on learning mechanisms contributes to explain whether and to what extent the patenting firm aims at influencing, or even determining, the industrial and technological trajectory.

Second, this study proposes an alternative theoretical viewpoint that merges two complementary perspectives involving knowledge and creativity aspects. In fact, this analysis prioritizes the industry-related dissemination processes, while previous studies disregarded the spillover properties within some particular technological areas. Last but not least, an additional and key theoretical contribution is the overarching approach embracing the macroeconomic and microeconomic implications altogether, even though research on such a dual aspect is still lacking. Such a gap proves the relevance of the theoretical contribution on macroeconomic and microeconomic perspectives related to citation flows. Hence, we fill this theoretical gap, as both of these perspectives are used in our study to highlight the advantages of knowledge dissemination and creative inter-nation pathways.

Thirdly, from a methodological standpoint, our approach avoids procedural and categorization issues, overcoming the limitations reported as patent office bias, and is bolstered even more by the dependability of the overall prediction % in Table 3a,c. We also develop and assess creativity through patent acquisitions.

We demonstrate that acquisitions of technology from abroad allow new owners to internalize patented knowledge, transform them into new patents, and stimulate their creativity, improving both firms’ and countries’ performances. Policymakers who are prepared to help the domestic healthcare sector should incentivize businesses to purchase patents from overseas in order to enrich organizational knowledge and create new technologies. Therefore, this study is potentially useful at the policy-making level for any country, especially those suffering from low levels of innovativeness in the healthcare sector, or those highly innovative in this field and willing to keep on succeeding in it. In brief, any country with a healthcare industry deemed as relevant to the national interest might benefit from this study. Regarding implications for businesses, managers are suggested to choose foreign technologies to acquire by looking at the technology’s age and the number of citations it has received. This way, a quicker internalization and exploitation through new patents can be achieved.

## Figures and Tables

**Table 1:** (a) Control variables of knowledge model; (b) Control variables of creativity model; (c) Descriptive statistics.

(**a**)				
**Variables’ Types**	**Variables’ Abbreviation**		**Variables’ Definition**	
**Technology**	TechAge		Technology Age	
	PatCiVal		Patent Citation Value	
**Firm**	FiSize		Firm Size	
	AppExp		Application Experience	
	R&D%		R&D Expenditure on Turnover	
	Prod		Production Value	
	NOPAT		Net Operating Profit After Taxes	
**Environment**	CoPaSt		Country Patent Stock	
(**b**)				
**Variables’ types**	**Variables’ abbreviation**		**Variables’ definition**	
**Technology**	PCD		Patented Creativity Degree	
**Firm**	FiSize		Firm Size	
	AppExp		Application Experience	
	R&D%		R&D Expenditure on Turnover	
	Prod		Production Value	
	NOPAT		Net Operating Profit After Taxes	
**Environment**	CoPaSt		Country Patent Stock	
(**c**)				
**Variables’ types**		**Variables’** **abbreviation**	**Mean**	**Std Dev.**
**Technology**		TechAge	0.752	1.783
		PatCiVal	0.511	1.563
**Firm**		FiSize	6.988	1.532
		AppExp	35,247	57,317.303
		R&D%	7.191	1.230
		Prod	5,713,960,000	52.043
		NOPAT	504,737,000	23.022
**Environment**		CoPaSt	51.439	9.679
**Technology**		PCD	19.622	13.455
**Firm**		FiSize	8.189	1.803
		AppExp	38,024	89,139.982
		R&D%	8.233	2.021
		Prod	4,713,960,000	43.455
		NOPAT	302,557,000	18.209
**Environment**		CoPaSt	21.110	8.903

**Table 2:** (a) Correlation test for the Knowledge Flow model; (b) Correlation test for the Creativity Flow model.

(**a**)									
	**IndVar**	**Tech** **Age**	**PatCiVal**	**FiSize**	**AppExp**	**R&D%**	**Prod**	**NOPAT**	**CoPaSt**
IndVar	1	0.756 **	0.883 **	0.096	−0.037	0.255	0.020	0.323	0.128
TechAge	0.756 **	1	0.807 **	0.070	−0.212	0.201	0.301	0.231	0.101
PatCiVal	0.883 **	0.807 **	1	0.053	−0.310	0.805 **	0.053	0.102	0.007
FiSize	0.096	0.070	0.053	1	0.653 **	0.405 **	0.135	0.055	0.455 **
AppExp	−0.037	−0.212	−0.310	0.653 **	1	0.502 **	0.232	0.320	0.354 **
R&D%	0.255	0.201	0.805 **	0.405 **	0.502 **	1	0.457 **	0.576 **	0.454 **
Prod	0.020	0.301	0.053	0.135	0.232	0.457 **	1	0.302	0.368 **
NOPAT	0.323	0.231	0.102	0.055	0.320	0.576 **	0.302	1	0.035
CoPaSt	0.128	0.101	0.007	0.455 **	0.354 **	0.454 **	0.368 **	0.035	1
(**b**)									
	IndVar	PCD	Country	FiSize	AppExp	R&D%	Prod	NOPAT	CoPaSt
IndVar	1	0.392 **	−0.023	0.201	0.230	0.207	0.153	0.245	0.039
PCD	0.392 **	1	−0.320	0.355	0.238	0.320	0.254	0.199	0.079
Country	−0.023	−0.320	1	0.097	0.202	0.756 **	0.201	0.103	0.236
FiSize	0.201	0.355	0.097	1	0.305 **	0.398 **	0.027	0.143	0.182
AppExp	0.230	0.238	0.202	0.305 **	1	0.572 **	0.151	0.278	0.575 **
R&D%	0.207	0.320	0.756 **	0.398 **	0.572 **	1	0.599 **	0.688 **	0.547 **
Prod	0.153	0.254	0.201	0.027	0.151	0.599 **	1	0.256	0.425 **
NOPAT	0.245	0.199	0.103	0.143	0.278	0.688 **	0.256	1	0.215
CoPaSt	0.039	0.079	0.236	0.182	0.575 **	0.547 **	0.425 **	0.215	1

** Correlation is significant at the 0.05 level (2-tailed).

**Table 3:** (a) Knowledge model: classification table; (b) Knowledge model: test results; (c) Creativity model: classification table; (d) Creativity model: test results.

(**a**)				
**Observed**		**Predicted**		
		**DepVar**		**Percentage Correct**
		**0.0000**	**1.0000**	
DepVar	0.0000	9018	0	100.0
	1.0000	5005	0	0.0
Overall Percentage				94.1
(**b**)				
		**Sig.**	**Log Likelihood**	**Wald Chi^2^**
Variable Model 1	IndVar	0.000	−73.572	23.441
	TechAge	0.001		
	PatCiVal	0.023		
	AppExp	0.037		
	R&D%	0.025		
	Prod	0.534		
	NOPAT	0.034		
	CoPaSt	0.770		
Variables Model 2	IndVar	0.000	−75.132	35.213
	TechAge	0.021		
	PatCiVal	0.038		
	FiSize	0.302		
	AppExp	0.338		
	R&D%	0.043		
	Prod	0.605		
	NOPAT	0.048		
	CoPaSt	0.900		
(**c**)				
**Observed**		**Predicted**		
		**DepVar**		**Percentage Correct**
		**0.0000**	**1.0000**	
DepVar	0.0000	10,473	0	100.0
	1.0000	3560	0	0.0
Overall Percentage				92.8
(**d**)				
		**Sig.**	**Log Likelihood**	**Wald Chi^2^**
Variables Model 1	IndVar	0.000	−86.225	19.723
	PCD	0.002		
	Country	0.041		
	AppExp	0.503		
	R&D%	0.032		
	Prod	0.557		
	NOPAT	0.021		
	CoPaSt	0.434		
Variables Model 2	IndVar	0.000	−83.223	21.710
	PCD	0.000		
	Country	0.078		
	FiSize	0.380		
	AppExp	0.791		
	R&D%	0.041		
	Prod	0.803		
	NOPAT	0.039		
	CoPaSt	0.575		

## Data Availability

Not applicable.
